# N-Heterocyclic Olefins as Organocatalysts for Polymerization: Preparation of Well-Defined Poly(propylene oxide)**

**DOI:** 10.1002/anie.201504175

**Published:** 2015-07-01

**Authors:** Stefan Naumann, Anthony W Thomas, Andrew P Dove

**Affiliations:** Department of Chemistry, University of Warwick, Materials and Analytical Science Building CV4 7AL Coventry (UK)

**Keywords:** N-heterocyclic olefins, organocatalysis, poly(propylene oxide), ring-opening polymerization

## Abstract

The metal-free polymerization of propylene oxide (PO) using a special class of alkene—N-heterocyclic olefins (NHOs)—as catalysts is described. Manipulation of the chemical structure of the NHO organocatalyst allows for the preparation of the poly(propylene oxide) in high yields with high turnover (TON>2000), which renders this the most active metal-free system for the polymerization of PO reported to date. The resulting polyether displays predictable end groups, molar mass, and a low dispersity (*Đ*_M_<1.09). NHOs with an unsaturated backbone are essential for polymerization to occur, while substitution at the exocyclic carbon atom has an impact on the reaction pathway and ensures the suppression of side reactions.

N-Heterocyclic olefins (NHOs), cyclic derivatives of ketene aminals (ene-1,1-diamines), comprise a group of highly polarized alkenes.[1] These compounds bear considerable electron density on their exocyclic carbon atom, which can be formally denoted as charge separation (Scheme 1 a). This unusual characteristic provides NHOs with remarkable properties, some of which have been exploited for the synthesis of NHO–metal complexes,[2] the formation of NHO–CO_2_ adducts,[3] or in Diels–Alder reactions.[4] Remarkably, Fürstner et al. found that even a very simple NHO (1,3-dimethyl-2-methyleneimidazoline Scheme 1 b) confers more electron density onto the metal center (see **A**) than typical N-heterocyclic carbenes (NHCs).[5] Moreover, coordination to the metal center was established to be end-on, in contrast to the commonly encountered side-on coordination of olefins, a clear testament to the strong polarization of the double bond. Furthermore, NHOs were also recently applied in Lewis pairs with aluminum-based co-catalysts for the polymerization of acrylates.[6]

**Scheme 1:**
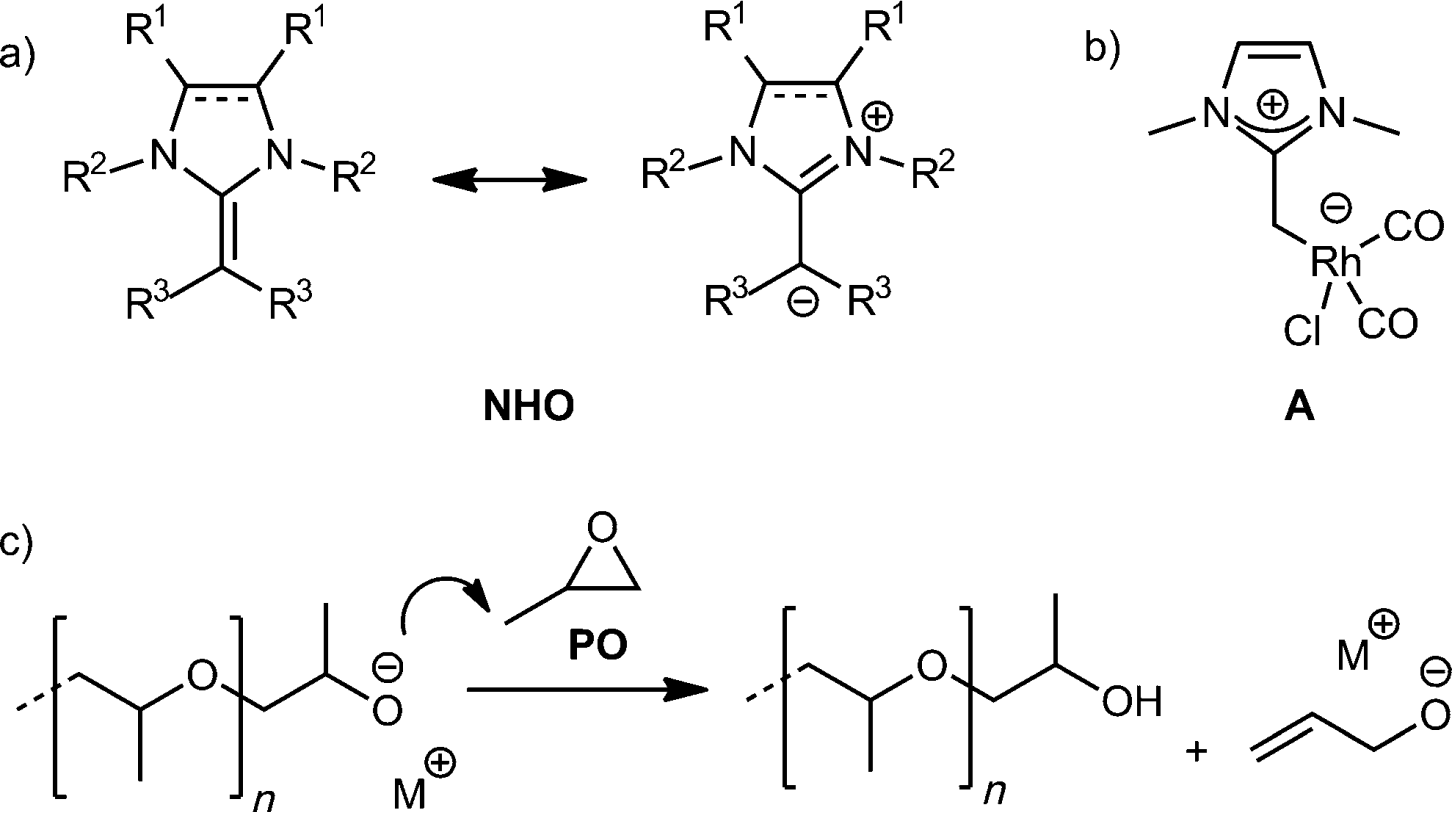
a) Mesomeric structures for NHOs; b) Fürstner′s NHO-rhodium complex, and c) transfer to monomer as a side reaction in PO polymerization. M^+^=organic or inorganic counterion.

The structural versatility of NHOs makes them exciting species that need to be explored more fully, the more so since they are closely related to so-called deoxy-Breslow intermediates, which are proposed to feature a prominent role in NHC-based organocatalysis.[7] Importantly, there are many ways to actively tune the degree of the double-bond polarization, which opens the possibility to design the reactivity of the NHO. This can be achieved simply by manipulation of the heterocycle. Charge separation is expected to be significantly favored with an unsaturated five-membered backbone, as the cyclic moiety can aromatize; this effectively “captures” the positive charge and maximizes the electron density on the exocyclic carbon atom. Likewise, positions R^1^–R^3^ can easily be varied by making use of well-established synthetic routes to form N-heterocycles. Although this structural diversity has so far only been explored to a limited degree, it potentially couples NHC-like adaptability with carbanionic reactivity. These characteristics suggest that NHOs will be ideally suited for application in a field where high nucleophilicity and basicity are essential.

Despite many recent advances in organocatalyzed polymerization, challenges remain to discover methods for the efficient polymerization of several important monomers. Propylene oxide, oligomers or polymers of which are mainly used in industry as long-chain polyether-polyol components for polyurethane formation,[8] is a prime example, as it has proven difficult to polymerize in the absence of metal activation. Organocatalysts alone typically display long reaction times, low turnover numbers (TON), or side reactions such as transfer to monomer (Scheme 1 c), which severely limit the accessible molecular weights and end-group fidelity.[9] Phosphazene bases have been successfully applied to polymerize ethylene oxide,[10, 11] but were reported to generate considerable levels of transfer to monomer when PO was used.[12] Perhaps more relevant to these investigations, Taton, Gnanou, and co-workers described an elegant, solvent-free process catalyzed by NHCs, which enabled the preparation of poly(propylene oxide) (PPO) with well-defined end groups and good control over the molecular weight.[13] However, yields were limited to 30–40 % at long reaction times (3 days) at 50 °C. NHC–CO_2_ adducts have also been used for the oligomerization of PO, although high catalyst loadings and high temperature are required.[14] Herein, we present the first application of NHOs as organic catalysts to overcome the difficulties in the metal-free synthesis of PPO.

NHOs **1**–**3** (Scheme 2 a) were prepared to determine the influence of the ring architecture on the reactivity. By using a convenient procedure to generate the target molecules from their precursor salts[3, 15] by deprotonation with KH,[1b, 2e, 5, 16] (Scheme S1), the NHOs were obtained after filtration and evaporation of the solvent, and could be used without further purification. Notably, NMR spectroscopic analysis revealed that the signals for the olefinic CH_2_ protons appear strongly shifted towards high field (*δ*=3.29–2.84 ppm, Figure S2), thus mirroring the increased electron density. NHO **3** displayed the strongest shift, in accordance with literature data,[1, 2] which can be attributed to the stronger contribution of the charge-separated mesomeric state that is a consequence of aromatization. The ring-opening polymerization (ROP) of PO was investigated in the bulk phase at 50 °C in the presence of benzyl alcohol (BnOH) as an initiator at only 0.1 % catalyst loading (NHO/BnOH/PO=1:10:1000). The initial results immediately revealed the impact of the N-heterocyclic ring system. While NHOs **1** and **2** did not yield any polymer, NHO **3** generated PPO with 57 % monomer conversion after 18.5 h, thus rendering this NHO more than four times more active than the benchmark NHC-based setup[13] (Table 1, entry 3). Moreover, when the reaction time was extended, near quantitative yields (96 %) were achieved (Table 1, entry 7). However, although analysis by gel-permeation chromatography (GPC) showed a very well-defined main peak (*Đ*_M_<1.07), a small high-molecular-weight impurity was also observed (Figure S3). Although minor, this observation suggested the presence of at least two different propagating species.

**Scheme 2:**
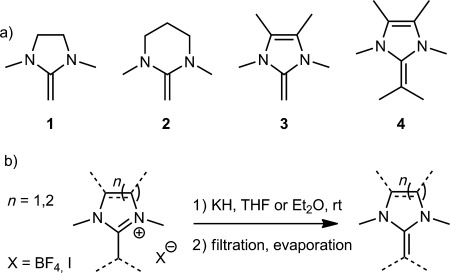
a) Catalysts prepared for this study and b) generalized synthetic procedure.

**Table 1:** Bulk polymerization of PO at 50 °C using NHOs 1–4

Entry	NHO	Time [h]	NHO/BnOH/PO	Conversion [%]^[a]^	*M*_n_ [g mol^−1^]^[b]^	*Đ*_M_
1	**1**	18.5	1:10:1000	0	–	–
2	**2**	18.5	1:10:1000	0	–	–
3	**3**	18.5	1:10:1000	57	3500	1.04
4	**4**	18.5	1:10:1000	43	3500	1.06
5	**3**	18.5	1:0:1000	<5	1800^[c]^	2.47
6	**4**	18.5	1:0:1000	0	–	–
7	**3**	68	1:10:1000	96	5600	1.06
8	**4**	68	1:10:1000	88	6700	1.04

[a] Calculated from ^1^H NMR spectra. [b] Determined by GPC analysis (CHCl_3_, polystyrene standards). [c] GPC chromatogram multimodal.

While NHC-catalyzed polymerization has been extended to the ROP of PO,[13] ethylene oxide,[17] lactones,[18] and siloxanes,[19] a mechanistic duality that involves both “basic” and “nucleophilic” mechanisms has been proposed.[7c, 20, 21] It is reasonable to assume that two different mechanistic pathways are also possible for NHO-catalyzed polymerization (Scheme 3): i) deprotonation of the initiator to induce a more classical anionic polymerization of PO, with the NHO as a non-innocent counterion that interacts with the propagating chain end (equivalent to the “basic” mechanism in NHC-catalyzed processes) and ii) nucleophilic attack of the NHO on the monomer with subsequent zwitterionic polymerization (equivalent to the “nucleophilic” mechanism in NHC-catalyzed processes). Liberation of the catalyst from the latter, proposed zwitterionic state, by nucleophilic substitution is strongly disfavored for NHCs, as shown by recent DFT calculations.[14] Such an elimination is even less likely for NHOs on account of the difference made by the additional carbon atom in the zwitterionic structure, hence trapping the NHO and preventing interconversion of the two propagating species, thus resulting in a multimodal molecular-weight distribution. We therefore reasoned that NHO **3** should also yield PPO in the absence of BnOH. Indeed, the subsequent bulk ROP of PO in the presence of only NHO **3** (Table 1, entry 5) resulted in isolation of a polymer, although in low yield (<5 %, Figure S4). Analysis of the resultant polymer by GPC revealed a multimodal distribution with a high molecular weight (number-average molecular weight, *M*_n_, up to 11 000 g mol^−1^).

**Scheme 3:**
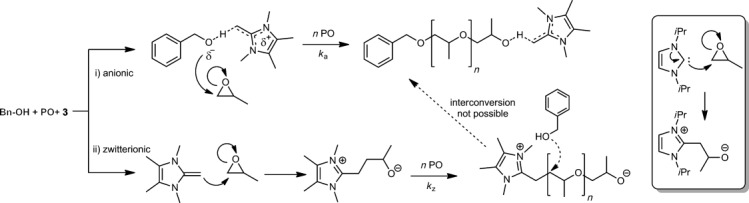
Major (anionic) and minor (zwitterionic) mechanisms proposed for the polymerization of PO using **3**. Box: Zwitterion derived from NHC.

To produce well-defined PPO, we sought to block the supposedly zwitterionic pathway by increasing the steric congestion of the catalytically active site. Hence, NHO **4** was synthesized, which bears two methyl groups on the exocyclic carbon atom. In addition to increased steric demands, this modification was proposed to enhance the basicity of the NHO on account of the generation of a latent tertiary carbanion (NHO **3** would generate a primary carbanion), and thus favor the anionic over the zwitterionic mechanism. Gratifyingly, under the same conditions outlined above, NHO **4** yielded PPO with low dispersity and a monomodal molecular weight distribution (Table 1, entry 4; Figure S3). Although this was coupled with a slightly decreased activity compared to NHO **3**, the molecular weight of the prepared polymer was fully predictable from the monomer/initator ratio as a consequence of the elimination of the side reactions (Figure S5). Importantly, NHO **4** did not yield any polymer when treated with PO in the absence of alcohol (Table 1, entry 6), thereby revealing a sharp contrast to typical NHC reactivity.[13] MALDI-ToF MS analysis of the PPO produced using NHO **4** underlined the well-defined nature of the polymerization (Figure 1). A single distribution was observed, with the major signal in the spectrum consistent with that calculated for a sodium-charged PPO initiated by benzyl alcohol. As supported by NMR spectroscopic analysis of the polymer, no allyl-terminated polymer chains were observed which indicates an absence of hydrogen abstraction as a relevant side reaction (Scheme 1).[9] Similar MALDI-ToF MS analysis of the PPO generated by NHO **3** additionally showed the expected high-molecular-weight impurity (Figure S6). These observations further corroborate the proposed polymerization mechanism.

**Figure 1:**
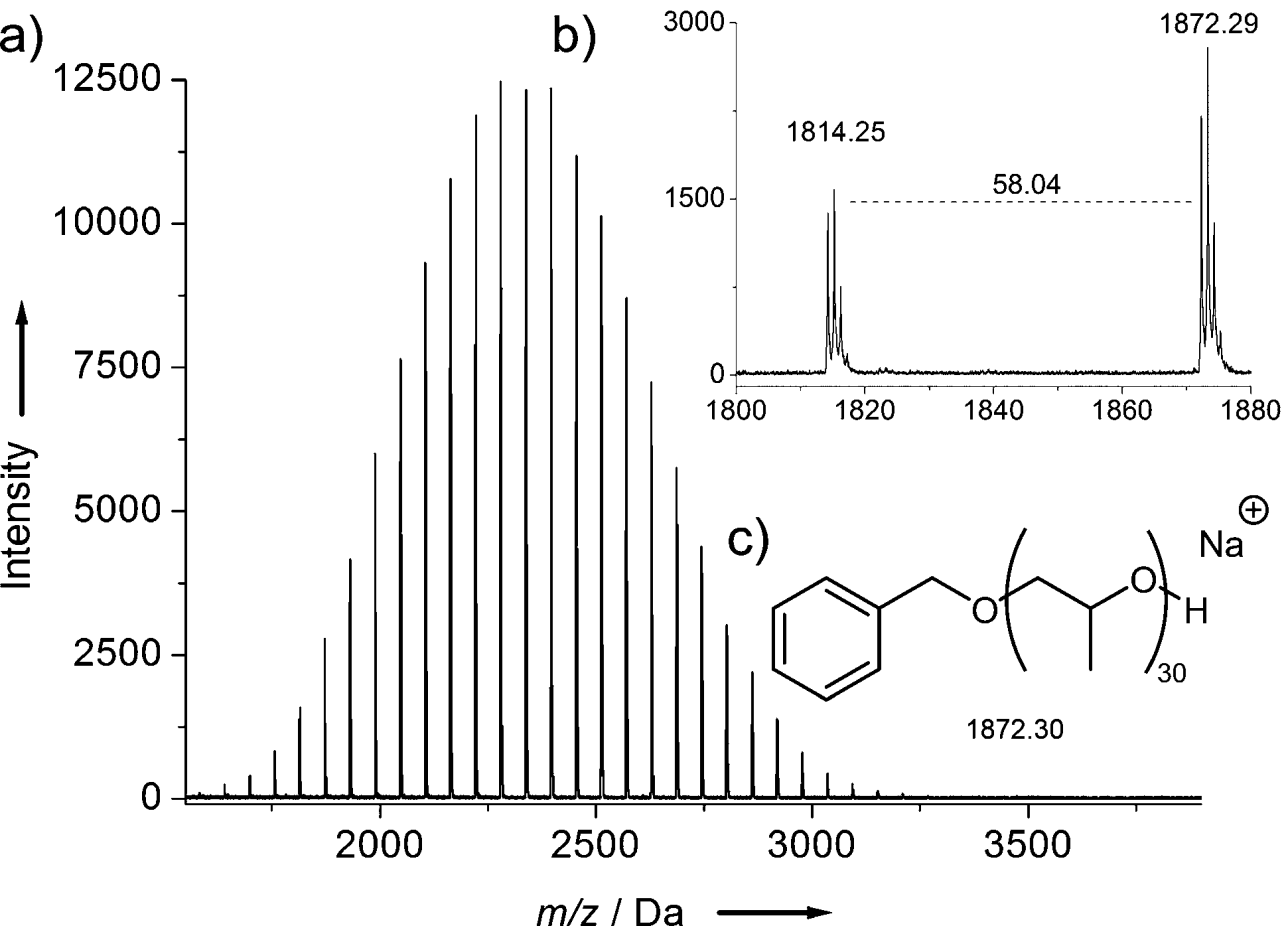
a) MALDI-ToF mass spectrum of PPO prepared by **4** (Table 1, entry 4), b) expansion, and c) calculated mass of sodium-charged PPO initiated by BnOH.

Clearly, the interaction of NHOs **1**–**4** with hydroxy groups is crucial for the success of the polymerization. To investigate these interactions further, a series of ^1^H NMR spectroscopy experiments was conducted (C_6_D_6_, BnOH/NHO=1:1, ambient temperature; Figures S7 and S8). The addition of NHO **1** to BnOH resulted in the disappearance of both the olefinic and -OH signals, which indicates some degree of interaction; the other signals showed little or no shift. However, this gradually changed when going from NHO **2** and **3** to NHO **4**. NHO **4** resulted in the appearance of a single broad resonance at *δ*=13.17 ppm, which most likely represents a significantly deshielded alcoholic proton.[22] In contrast, a broad signal is observed at *δ*=4.65 ppm in the presence of NHO **3**. Most likely, this series of spectra show BnOH–NHO complexes in different stages of the deprotonation process: the stronger base, **4**, is able to abstract the proton of the alcohol to a higher degree than **3**, the weaker base. Consistently, the methylene unit Ar-*CH_2_*-OH is shifted stepwise towards low field (*δ*=4.44, 4.67, 5.05, and 5.19 ppm for NHOs **1**–**4**, respectively) and likewise the convoluted aromatic region of BnOH becomes increasingly differentiated. Both observations are again in line with increasing deprotonation, which will increase the negative charge on the oxygen atom (and therefore the shielding of the adjacent methylene moiety) and also strengthen the inductive effect on the aromatic ring. This outcome strongly emphasizes the difference in the reactivity of the studied NHOs. Since NHOs **1** and **2** do not yield PPO, but NHOs **3** and **4** do, these interactions seem critical to defining the reactivity that has to be surpassed to induce polymerization.

Once it had been established that catalyst **4** was able to produce highly defined and monomodal PPO, further investigations of the key properties of the polymerization of PO catalyzed by this NHO were undertaken. Identical but independent polymerizations were stopped after different reaction times, to correlate the conversion and molecular weight. Notably, this resulted in a perfectly linear relationship (Figure 2) over the range 1700–6700 g mol^−1^, while maintaining low dispersity (*Đ*_M_<1.09). Additionally, with the proposed role of the NHO as the catalyst and BnOH as the initiator, a change in the ratios was anticipated to have distinct consequences on the molecular weight of the resultant PPO. Although a higher loading of NHO **4** at constant BnOH/PO resulted in a more rapid polymerization, the molecular weight remained within the expected range (Table 2, entry 1; compare Table 1, entry 4 and Figure S5). In a complementary manner, doubling the amount of BnOH (to target a degree of polymerization (DP) of 50) halved the resulting molecular weight, while a target DP=200 yielded PPO with a molecular weight greater than 10 000 g mol^−1^ (Table 2, entries 2 and 4). When the target DP was increased further to 300, the monomer conversion dropped significantly (Table 2, entry 5) and remained below the level of the lower DP polymerizations even when the reaction time was extended (Table 2, entry 6). Nonetheless, a molecular weight of 12 000 g mol^−1^ was achieved in this way, which is atypically high for PPO derived by organocatalysis. These results suggest that the presence of BnOH is important to retain high catalyst activities, possibly as a result of increased overall solution polarity and lower viscosity of the solution as a result of lower molecular weight polymers. Importantly, not only is the rate of monomer consumption influenced, but also the occurrence of transfer to monomer. While this is virtually absent for NHO/BnOH ratios of 1:10 or higher, it gets more noticeable for low BnOH loadings (1:5 or lower, DP>200) (Figures S9 and S10), most likely a consequence of the decreased ratio of alcohol to monomer leading to increased competition for proton abstraction from PO in preference to the alcohol.

**Figure 2:**
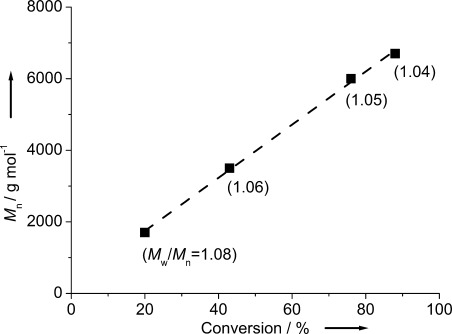
Monomer conversion versus molecular weight (*M*_n_, GPC). **4**/BnOH/PO=1:10:1000, 50 °C, bulk.

**Table 2:** Bulk polymerization of PO at 50 °C using NHO 4

Entry	Time [h]	NHO/BnOH/PO	Conversion [%]^[a]^	*M*_n_ [g mol^−1^]^[b]^	*Đ_M_*
1	18.5	2:10:1000	66	5100	1.04
2	68	1:20:1000	92	3900	1.06
3	68	1:10:1000	88	6700	1.04
4	68	1:5:1000	84	10 100	1.05
5	68	1:3.3:1000	59	9300	1.06
6	138	1:3.3:1000	75	12 000	1.06
7	113	1:30:3000	73	5800	1.04
8	113	1:100:10 000	26	2100	1.06

[a] Calculated from ^1^H NMR spectra. [b] Determined by GPC analysis (CHCl_3_, polystyrene standards).

Finally, to further determine the limits of the ROP of PO by NHO **4**, reactions with very low NHO loadings were conducted, using the aforementioned optimized conditions of PO/BnOH=100:1. Interestingly, with 3000 equivalents of monomer (Table 2, entry 7), a relatively high conversion of 73 % was achieved. This corresponds to a TON of about 2200, unrivalled by competing organocatalytic systems to date. A further increase to 10 000 equivalents of PO (0.01 % NHO) at the same polymerization time resulted in a further increased TON of 2600 (at 26 % monomer conversion), thereby underlining the robustness of the catalyst and the absence of relevant poisoning by impurities. In both cases, transfer to monomer was insignificant (Figure S11).

In conclusion, we have reported the first example of an NHO-catalyzed organopolymerization. Through manipulation of the NHO structure we have shown that the nature of the heterocyclic ring is a key factor in determining the activity in the polymerization of PO. Imidazolium-based catalysts show a high performance, while their saturated five- and six-membered counterparts do not polymerize PO at all. Furthermore, it was demonstrated that the exocyclic carbon atom is a decisive tuning site of the catalysts, and can be used to suppress undesired side reactions. Increased steric congestion and basicity, as present in NHO **4**, enabled the synthesis of well-defined PPO in a highly controlled manner, while at the same time, to the best of our knowledge, this NHO emerged as the most active organocatalyst for PO polymerization reported to date. The distinct NHO carbanionic reactivity can be expected to allow access to further highly challenging fields in organopolymerization, especially since the catalysts can be readily tailored, as shown in this study.

## Supporting Information

As a service to our authors and readers, this journal provides supporting information supplied by the authors. Such materials are peer reviewed and may be re-organized for online delivery, but are not copy-edited or typeset. Technical support issues arising from supporting information (other than missing files) should be addressed to the authors.

miscellaneous_information
