# Supplementary material for: N-Heterocyclic Olefins as Organocatalysts for Polymerization: Preparation of Well-Defined Poly(propylene oxide)
Source: Angew Chem Int Ed Engl. 2015 Jul 1;54(33):9550–4. doi: 10.1002/anie.201504175 (PMC4539597; doi:10.1002/anie.201504175)
Supplement: Supplementary file 1 — miscellaneous_information [file anie0054-9550-sd1.pdf]

Angewandte Chemie
Eine Zeitschrift der Gesellschaft Deutscher Chemiker

Supporting Information

# N-Heterocyclic Olefins as Organocatalysts for Polymerization: Preparation of Well-Defined Poly(propylene oxide)**

*Stefan Naumann,* Anthony W. Thomas, and Andrew P. Dove**

anie_201504175_sm_miscellaneous_information.pdf

# Contents

Experimental ........ 1
Materials and Synthesis of NHOs ........ 1
General Preparation of NHOs 1-3 ........ 2
Polymerization of PO ........ 3
Characterization and Analysis ........ 3
Other Supporting Data ........ 4
References ........ 11

# Experimental

### *Materials and Synthesis of NHOs*

± Propylene Oxide (PO, Aldrich) was dried by stirring with $CaH_2$ under nitrogen atmosphere overnight. The monomer was then distilled thermally under inert conditions, degassed and stored in a glove box (*UniLab*, MBraun, Germany) at -36 °C. Special care should be taken as PO is very volatile (b.p. = 34 °C), toxic and carcinogenic. Benzylic alcohol (BnOH) and deuterated benzene ($C_6D_6$) were dried over sodium, distilled under nitrogen, degassed and stored over molecular sieves at -36 °C in a glove box. All other chemicals were obtained from Sigma-Aldrich and used as received. NHOs **1-4** were prepared following known literature procedures (S1).[i-vi] The precursor salts were synthesized by cyclization of diamines with a suitable orthoester (precursors to **1** and **2**),[i] by methylation of a commercially available imidazole (precursor to **3**) or *via* a Radziszewski-type reaction (precursor to **4**).[ii] To prepare the NHOs **1-3**, a generalized procedure was applied (see below). The identity of target compounds **1-4** was confirmed by $^{1}H$ and $^{13}C$ NMR spectroscopy and they were stored in a glove box at -36 °C. For full characterization of **1**,[iii] **2**,[iv] and **3**[v] and for experimental details and characterization of **4**[vi] see the cited literature.

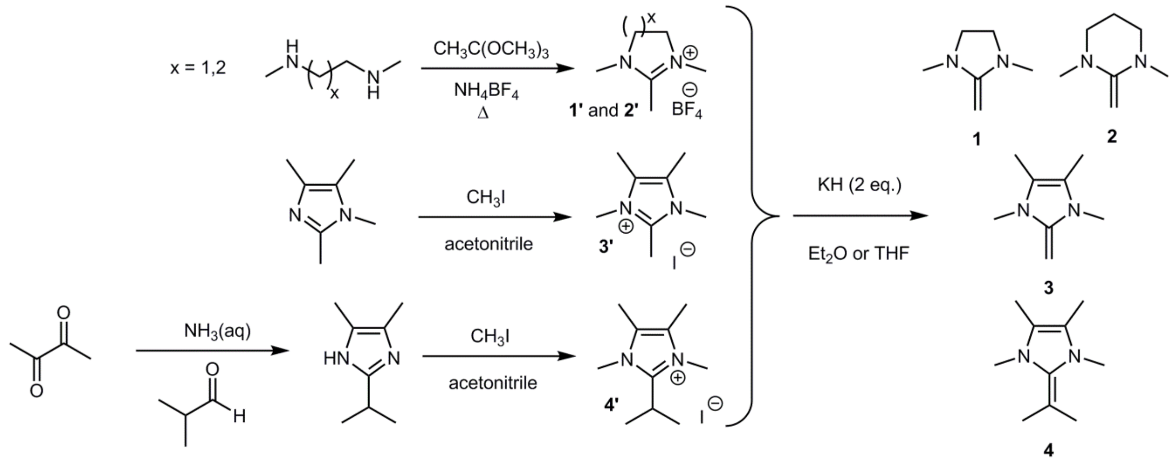


**Scheme S1**. Schematic preparation of the NHO catalysts used in this study.

### *General Preparation of NHOs 1-3*

Potassium hydride (KH, 770 mg, 19 mmol) was suspended in dry diethyl ether (25 mL) in a Schlenk flask. Precursor salts **1'**, **2'** or **3'** were slowly added (0.5 equivalents, mild gas development). The reaction was then stirred for 48 h at ambient temperature, under exclusion of light. This was followed by evaporation of the solvent *in vacuo* at 0 °C. The reaction vessel was subsequently transferred into the glove box, where the residues were extracted with pentane and filtrated to yield clear solutions. Compounds **1-3** were received after removal of the pentane under vacuum (0 °C).

**1,3-dimethyl-2-methyleneimidazolidine (1)**: 815 mg (liquid, 73%) yield. $^{1}$**H NMR** ($C_6D_6$, 400 MHz, 298 K): $\delta$ = 3.14 (s, 2H), 2.61 (s, 4H), 2.43 (2, 6H) ppm. $^{13}$**C NMR** ($C_6D_6$, 100 MHz, 298 K): $\delta$ = 52.3, 50.3, 35.6 ppm.

**1,3-dimethyl-2-methylenetetrahydropyrimidine (2)**: 900 mg (liquid, 71%) yield. $^{1}$**H NMR** ($C_6D_6$, 400 MHz, 298 K): $\delta$ = 3.29 (s, 2H), 2.56 (t, 4H), 2.48 (s, 6H), 1.62 (qt, 2H) ppm. $^{13}$**C NMR** ($C_6D_6$, 100 MHz, 298 K): $\delta$ = 165.4, 63.1, 50.6, 40.3, 24.9 ppm.

**2-methylene-1,3,4,5-tetramethylimidazoline (3)**: 900 mg (solid, 69%) yield. $^{1}$**H NMR** ($C_6D_6$, 400 MHz, 298 K): $\delta$ = 2.84 (s, 2H), 2.62 (s, 6H), 1.49 (s, 6H) ppm. $^{13}$**C NMR** ($C_6D_6$, 100 MHz, 298 K): $\delta$ = 40.5, 29.3, 8.6 ppm.

***Polymerization of PO***

For a typical polymerization experiment, PO was mixed with BnOH and added to NHO 4. The colorless mixture was transferred to a sealed glass vessel (50 mL ampoule, dried overnight at 160 °C) and submerged in a pre-heated oil-bath (50 °C). During the course of the reaction the polymerization turned a pale yellow. The polymerization was quenched by evacuation to remove excess PO (1-2 h). As a consequence of the volatility of PO, the degree of polymerization and conversion were calculated by $^1$H NMR spectroscopy ($CDCl_3$), using the ratio of the methylene unit of the initiator ((Ar-$\underline{CH_2}$-O-) at $\delta$ = 4.5 ppm) versus the polymer signals at $\delta$ = 3.4 ppm and $\delta$ = 1.1 ppm. The PPO was stored under ambient conditions prior to further analysis during which time the pale yellow discoloration disappeared.

***Characterization and Analysis***

A *Bruker DPX 400* spectrometer was used for recording of proton ($^1$H) and carbon ($^{13}$C) NMR spectra. All chemical shifts are reported in parts per million (ppm), relative to reference peaks for proton and carbon NMR experiments ($CDCl_3$: $\delta$ = 7.26/77.16 ppm, $C_6D_6$: $\delta$ = 7.16/128.06 ppm). The molecular weight of the PPO was determined *via* gel permeation chromatography (GPC) using a system consisting of an *Agilent 390-MDS* and PLgel Mixed D-type columns in combination with a refractive index detector. Samples were run in chloroform (0.5% $NEt_3$, 40 °C, 1 mL min$^{-1}$) and a calibration from polystyrene standards was applied. Sample concentration was 7 mg mL$^{-1}$. MALDI-ToF (matrix-assisted laser desorption ionization-time of flight) mass spectrometry measurements were performed on a *Bruker Autoflex Speed* TOF/TOF mass spectrometer using a nitrogen laser delivering 2 ns pulses at 337 nm with positive ion ToF detection performed using an accelerating voltage of 25 kV. Trans-2-[3-(4-tertbutylphenyl)-2-methyl-2-propylidene]malonitrile (DCTB) was applied as a matrix (0.2 μL of a 10 g L$^{-1}$ solution in tetrohydrofuran), with sodium trifluoroacetate used as a cationization agent (0.1 μL of a 10 g L$^{-1}$ solution in tetrohydrofuran). Analyte (0.1 μL of a 1 g L$^{-1}$solution in tetrohydrofuran) was applied in between separate loadings of DCTB and sodium trifluoroactetate, with solvent being allowed to evaporate between applications, to form a thin matrix-analyte-matrix film. All samples were measured in reflectron mode and calibrated against monodisperse *Polymer Factory* SpheriCal® dendritic standards (calibration range = 500-7000 Da).

# Other Supporting Data

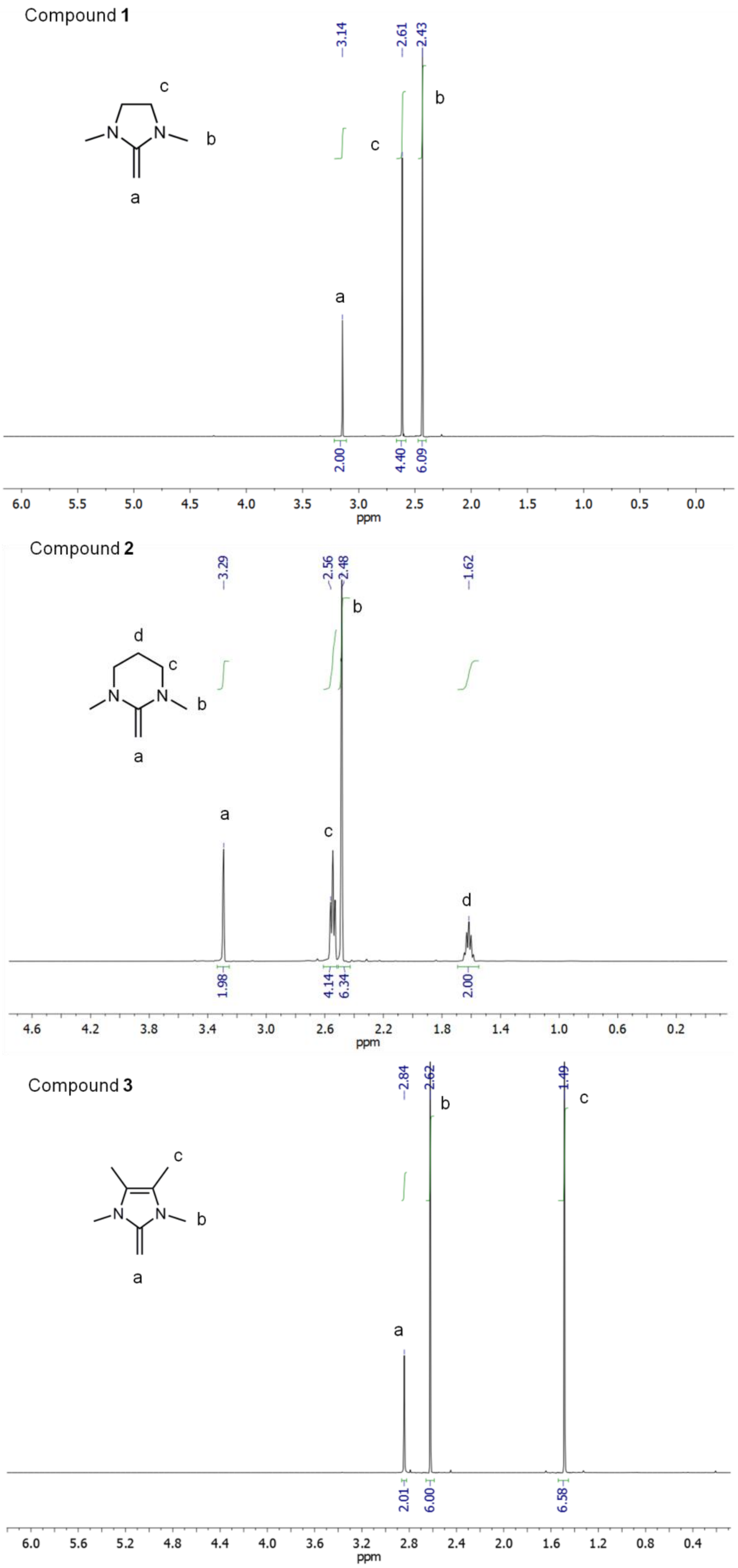


**Figure S2.** $^{1}H$ NMR spectra of NHOs **1-3** ($C_6D_6$, 400 MHz, 298K). Note the high field shift of the olefinic protons (a).

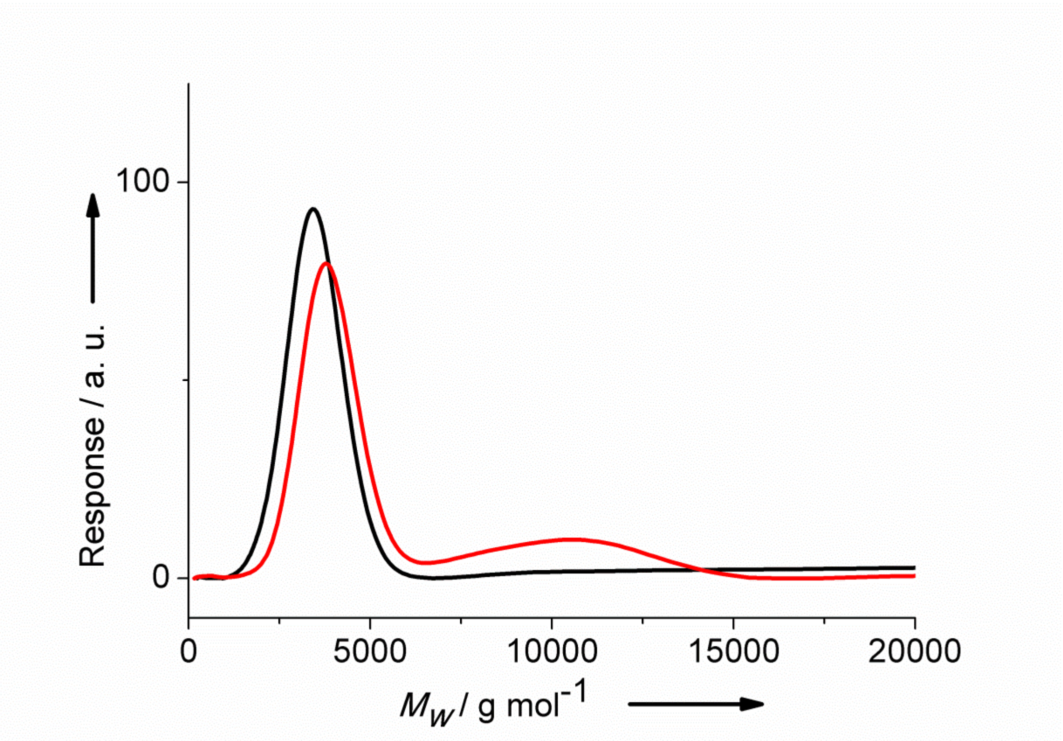


**Figure S3.** GPC chromatogram of PO polymers (Table 1, entries 3 and 4) by **3** (red) and **4** (black). Note the higher molecular weight impurity when **3** is used as catalyst.

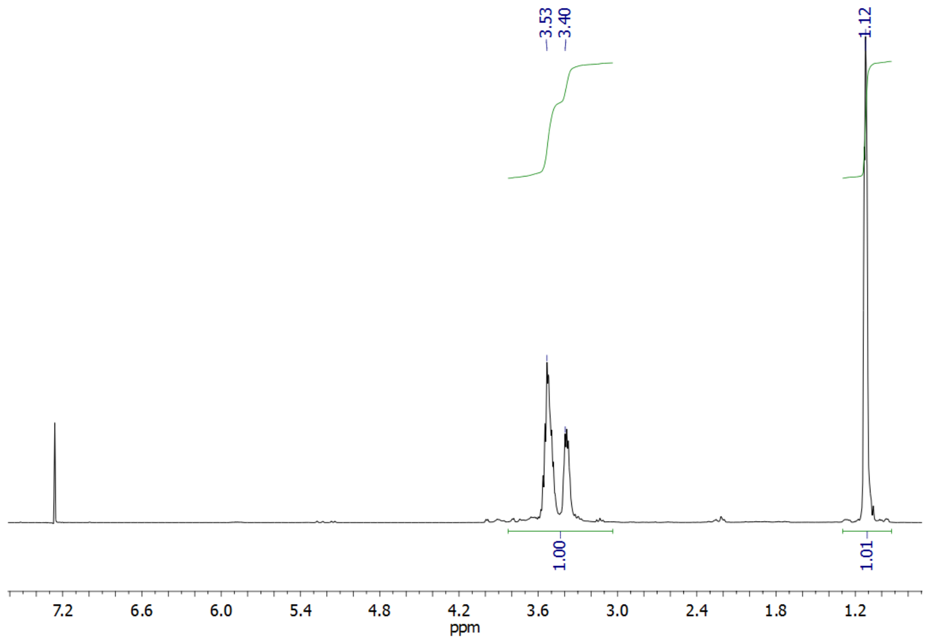


**Figure S4.** $^{1}$H NMR spectrum ($CDCl_3$, 400 MHz, 298K) of PPO derived from **3**/PO in the absence of BnOH (Table 1, entry 5).

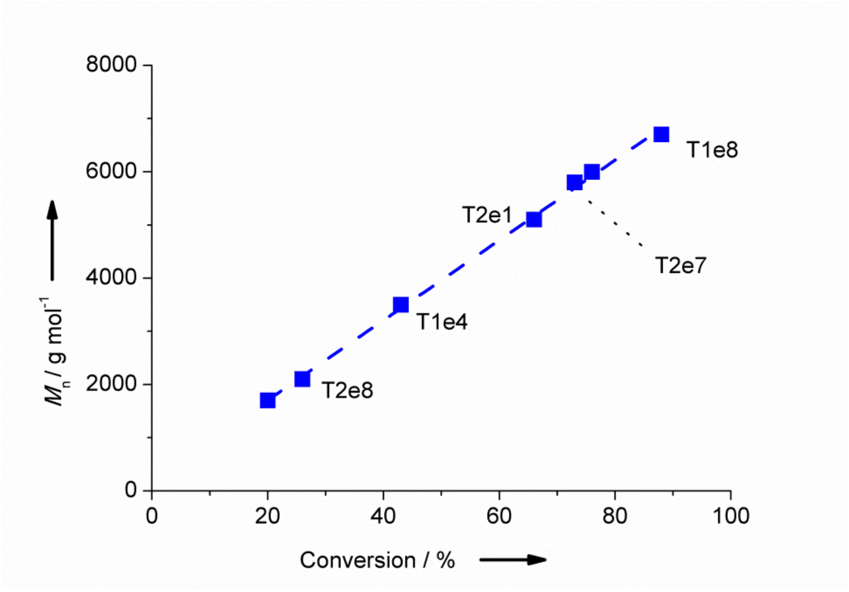


**Figure S5.** Correlation of number-average molecular weight ($M_n$) *versus* conversion for all experiments with BnOH:PO = 1:100 (DP = 100) presented in this work. Note that the linear relationship is valid for a broad range of catalyst concentrations (1:500-1:10 000) and reaction times (8-113 h). T = table, e = entry.

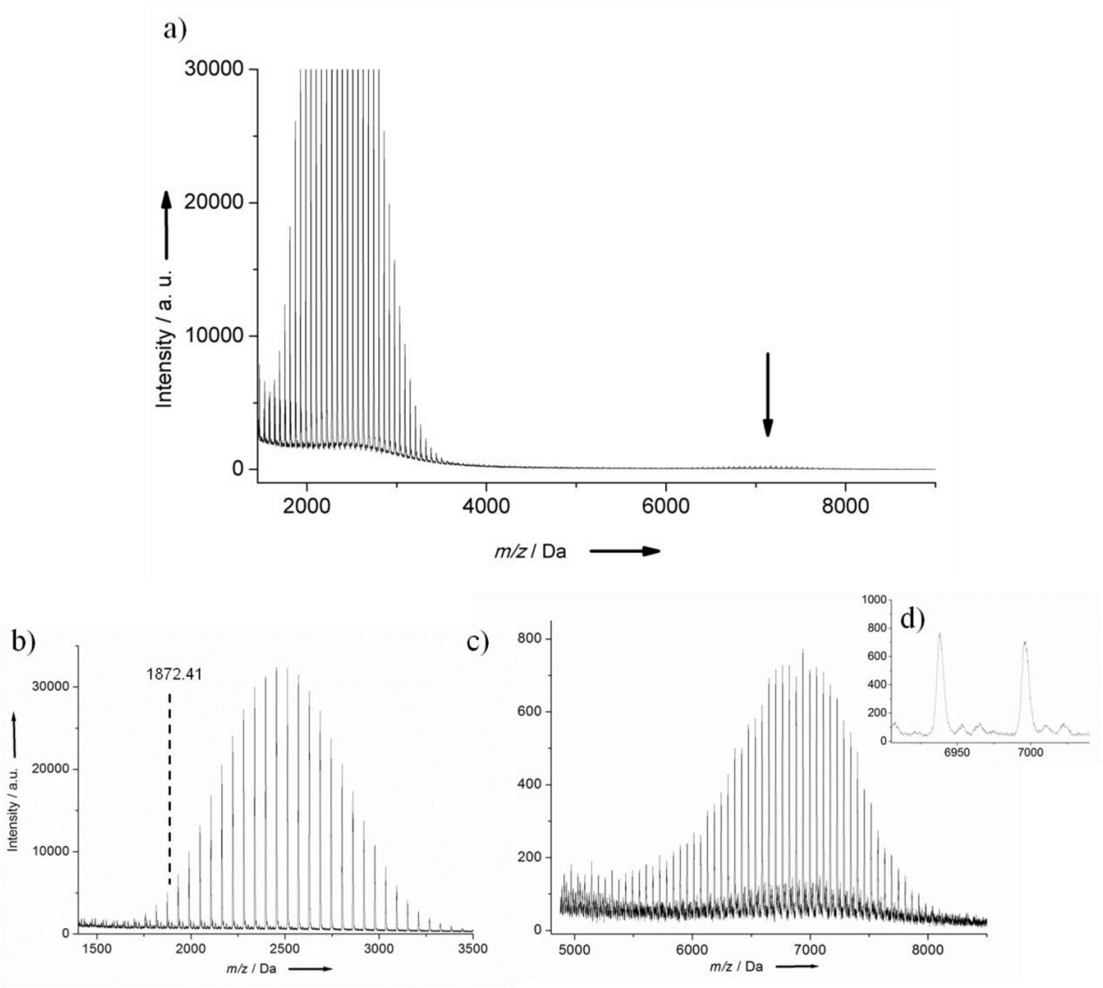


**Figure S6.** MALDI-ToF mass spectra of PPO prepared by the action of catalyst **3** (Table 1, entry 3). (a) full range spectrum, showing the dominating major distribution and the minor high molecular weight impurity (arrow, compare Figure S3). (b) Separate measurement for the low molecular weight region with denoted mass peak (compare Figure 3) and (c) the high molecular weight range with (d) expansion.

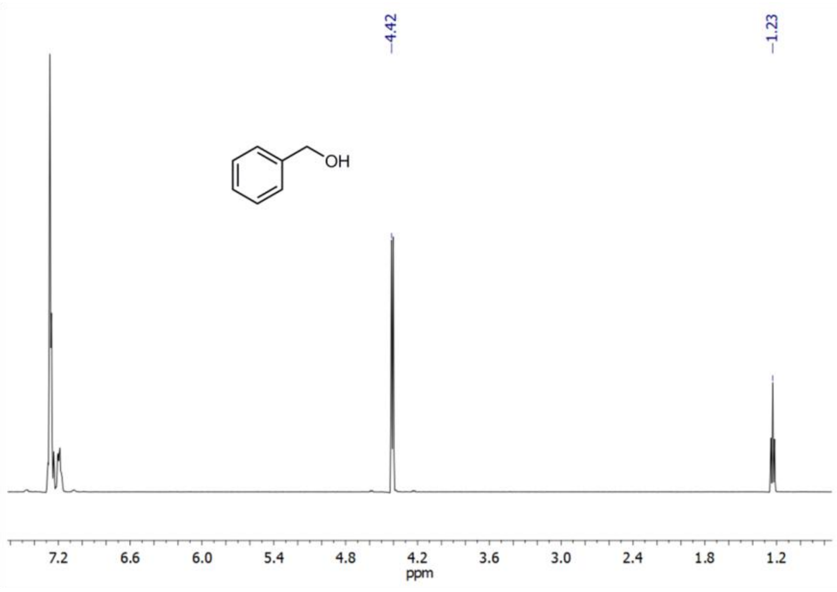


**Figure S7.** $^{1}$H NMR of benzyl alcohol ($C_6D_6$, 400 MHz, 298K). Note the typical convoluted aromatic region, the shift of the methylene unit at $\delta$ = 4.42 ppm and the –OH signal at $\delta$ = 1.23 ppm.

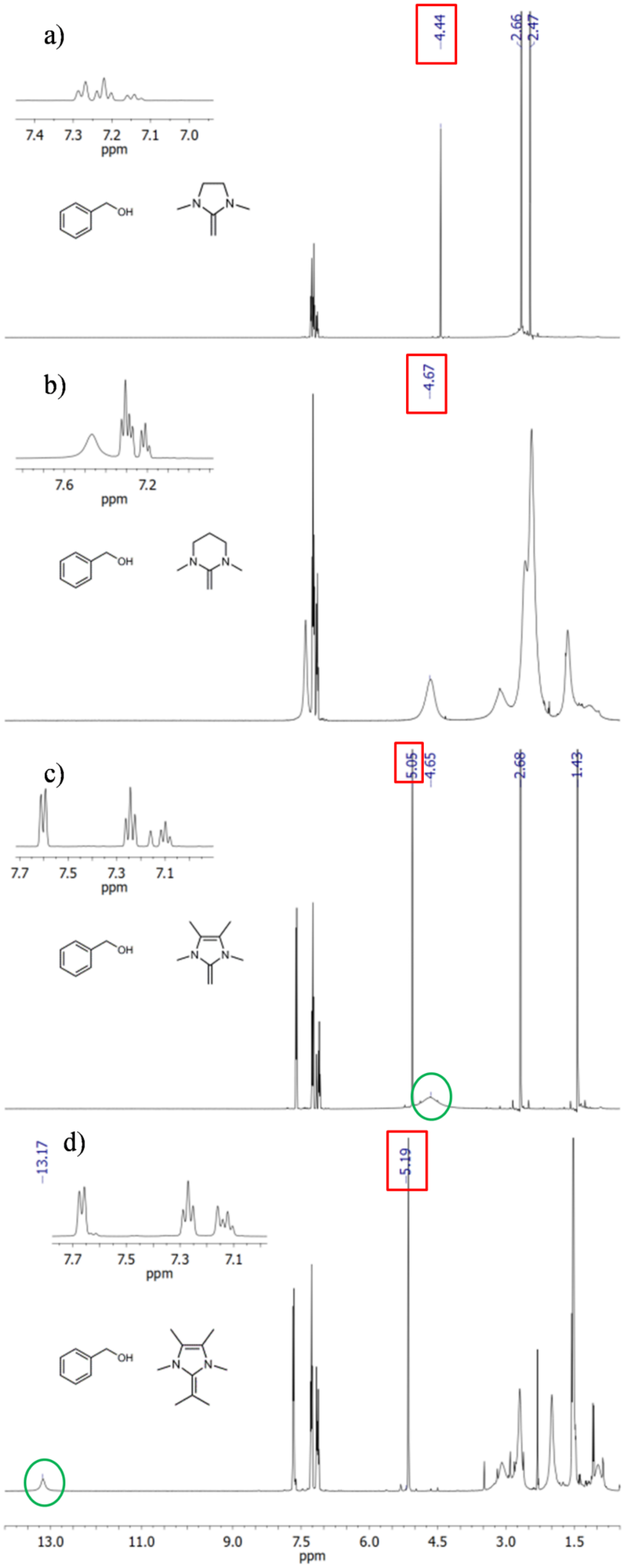


**Figure S 8.** $^{1}$H NMR spectra of BnOH combined with NHOs **1-4** (1:1, $C_6D_6$, 400 MHz, 298 K). Note the gradual shift of the methylene unit of BnOH (red), which is virtually unchanged when in the presence of **1** (a), but appears at $\delta$ = 5.19 ppm when **4** is applied (d). Likewise, the aromatic region gets more resolved, strongest for NHOs **3** and **4**, both of which polymerize PO. Notably in both latter cases distinct broad signals appear (green), most likely representing the deshielded hydroxylic proton that is partially abstracted by the strongly basic NHO. The effect is much more prominent for **4** ($\delta$ = 13.17 ppm, d)) than for **3** ($\delta$ = 4.65 ppm, c)), reflecting supposedly the difference in basicity for both catalysts.

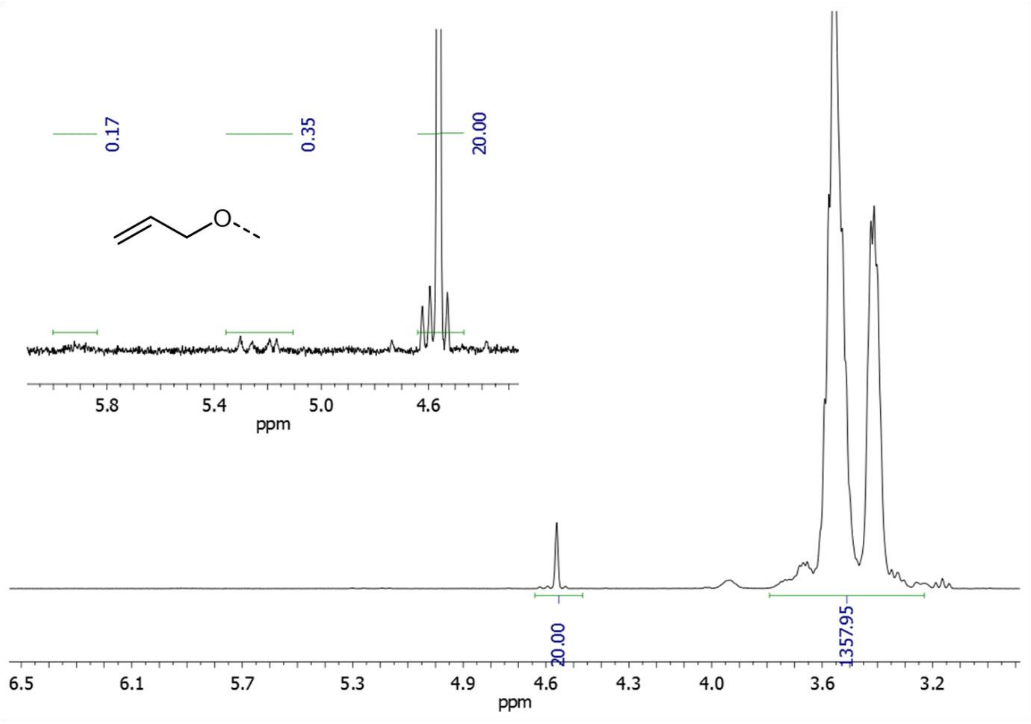


**Figure S9.** $^{1}$H NMR spectrum ($CDCl_3$, 400 MHz, 298K) of PPO derived from the action of **4**/BnOH/PO = 1/20/1000. The insert shows the very weak presence of allylic protons from transfer-to-monmer (Table 2, entry 2).

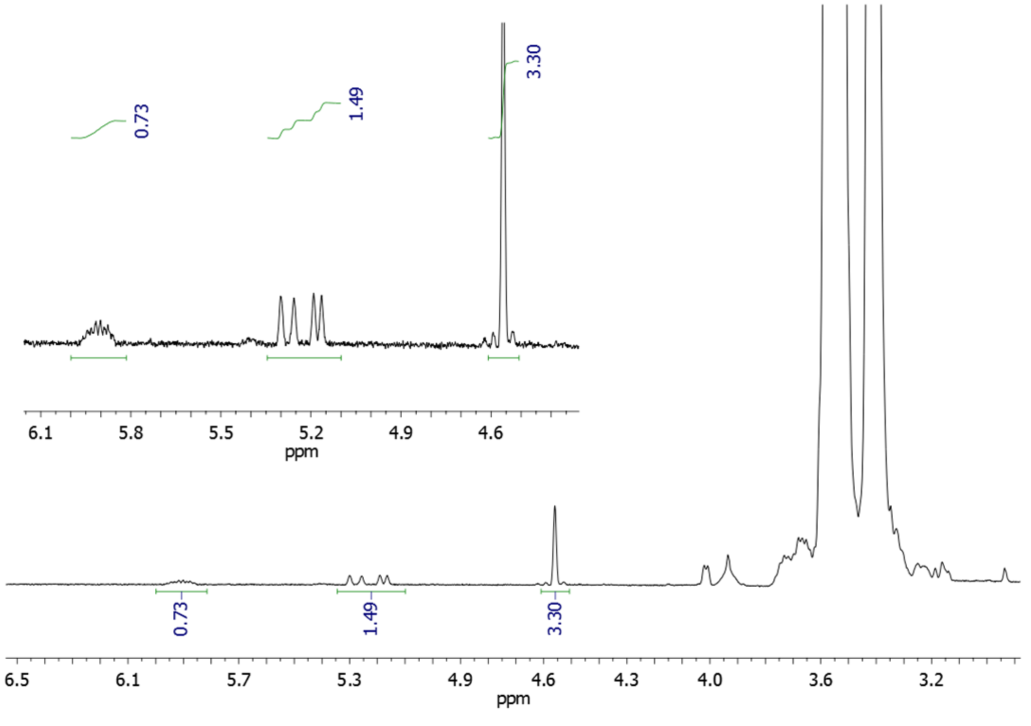


**Figure S10.** $^{1}$H NMR spectrum ($CDCl_3$, 400 MHz, 298K) of PPO derived from the action of **4**/BnOH/PO = 1/3.3/1000. The insert shows the increased presence of allylic protons from transfer-to-monomer at low alcohol loadings (Table 2, entry 5).

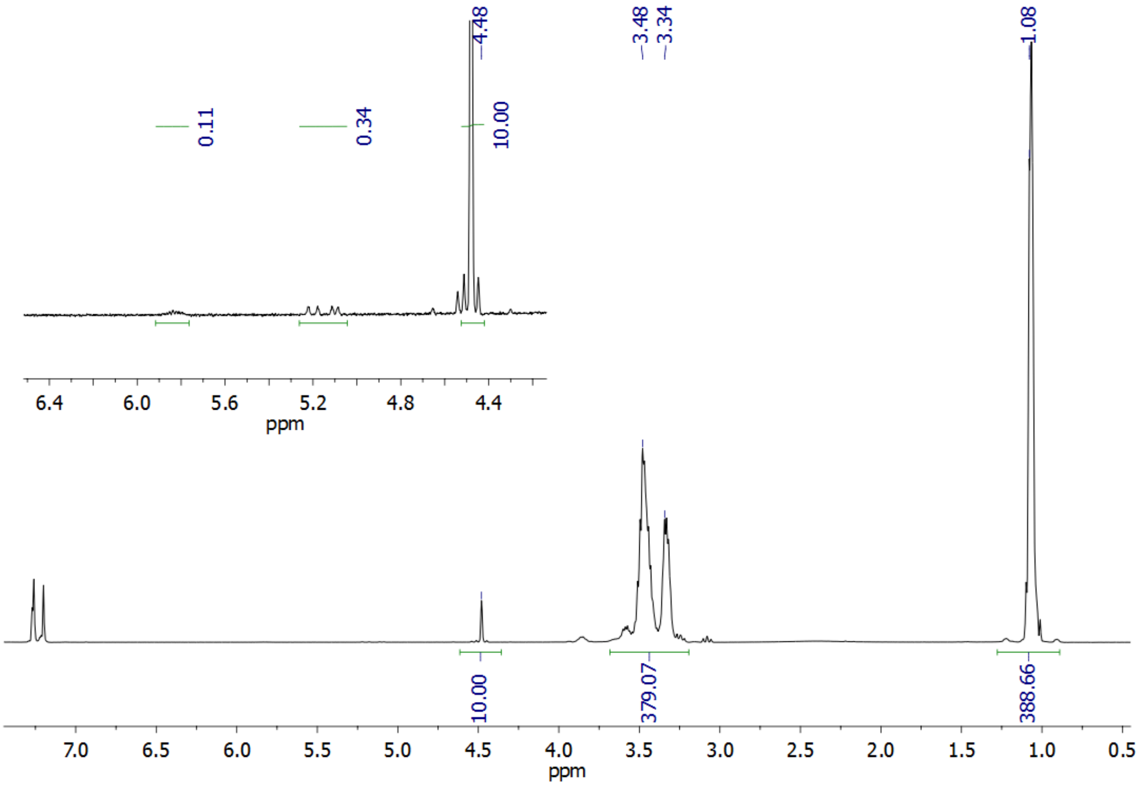


**Figure S11.** $^{1}$H NMR spectrum ($CDCl_3$, 400 MHz, 298K) of PPO derived from the action of **4**/BnOH/PO = 1/100/10 000 (Table 2, entry 8).

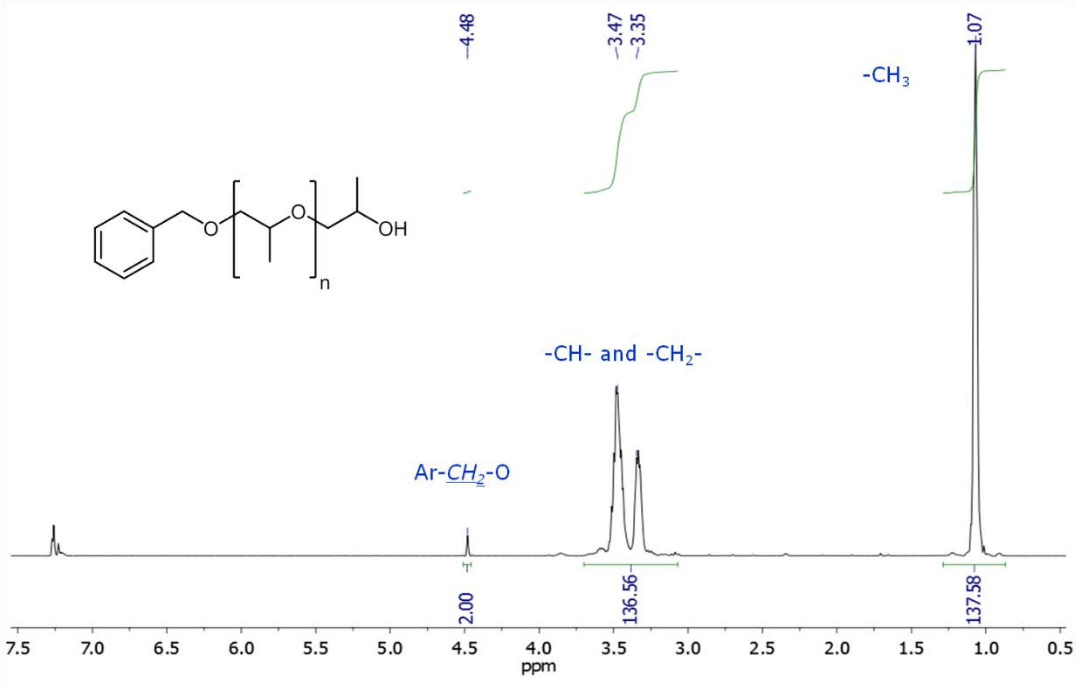


**Figure S12.** $^{1}$H NMR spectrum ($CDCl_3$, 400 MHz, 298K) of typical PPO as received by action of NHO/BnOH (details: Table 1, entry 4).

## References

[i] S. Saba, A. Brescia, M. K. Kaloustian, *Tetrahedron Lett.* **1991**, *32*, 5031–5034.

[ii] A. R. Chianese, B. M. Zeglis, R. H. Crabtree, *Chem. Commun.* **2004**, 2176–2177.

[iii] Gruseck, U.: Heuschmann, M., *Chem. Ber.* **1987**, *120*, 2053–2064.

[iv] G. Ye, W. P. Henry, C. Chen, A. Zhou, C. U. Pittman, *Tetrahedron Lett.* **2009**, *50*, 2135–2139.

[v] N. Kuhn, H. Bohnen, J. Kreutzberg, D. Bläser, R. Böse, *J. Chem. Soc., Chem. Commun.* **1993**, 1136-1137.

[vi] S. Kronig, P. G. Jones, M. Tamm, *Eur. J. Inorg. Chem.* **2013**, *2013*, 2301–2314.
